# Increased mitochondrial mutation heteroplasmy induces aging phenotypes in pluripotent stem cells and their differentiated progeny

**DOI:** 10.1111/acel.14402

**Published:** 2024-12-16

**Authors:** Amy R. Vandiver, Alejandro Torres, Amberly Sanden, Thang L. Nguyen, Jasmine Gasilla, Mary T. Doan, Vahan Martirosian, Austin Hoang, Jonathan Wanagat, Michael A. Teitell

**Affiliations:** ^1^ Division of Dermatology, Department of Medicine, David Geffen School of Medicine University of California at Los Angeles Los Angeles California USA; ^2^ Veterans Administration Greater Los Angeles Healthcare System Los Angeles California USA; ^3^ Department of Pathology and Laboratory Medicine, David Geffen School of Medicine University of California at Los Angeles Los Angeles California USA; ^4^ Department of Biology California State University Northridge California USA; ^5^ Department of Molecular and Medical Pharmacology, David Geffen School of Medicine University of California at Los Angeles Los Angeles California USA; ^6^ Division of Geriatrics, Department of Medicine, David Geffen School of Medicine University of California at Los Angeles Los Angeles California USA; ^7^ Molecular Biology Institute University of California at Los Angeles Los Angeles California USA; ^8^ Department of Bioengineering, California Nano Systems Institute, and Broad Center for Regenerative Medicine and Stem Cell Research University of California at Los Angeles Los Angeles California USA; ^9^ Department of Pediatrics, David Geffen School of Medicine University of California at Los Angeles Los Angeles California USA; ^10^ Jonsson Comprehensive Cancer Center, David Geffen School of Medicine University of California at Los Angeles Los Angeles California USA

**Keywords:** aging, iPSC, mitochondria, mtDNA mutation

## Abstract

The mitochondrial genome (mtDNA) is an important source of inherited extranuclear variation. Clonal increases in mtDNA mutation heteroplasmy have been implicated in aging and disease, although the impact of this shift on cell function is challenging to assess. Reprogramming to pluripotency affects mtDNA mutation heteroplasmy. We reprogrammed three human fibroblast lines with known heteroplasmy for deleterious mtDNA point or deletion mutations. Quantification of mutation heteroplasmy in the resulting 76 induced pluripotent stem cell (iPSC) clones yielded a bimodal distribution, creating three sets of clones with high levels or absent mutation heteroplasmy with matched nuclear genomes. iPSC clones with elevated deletion mutation heteroplasmy show altered growth dynamics, which persist in iPSC‐derived progenitor cells. We identify transcriptomic and metabolic shifts consistent with increased investment in neutral lipid synthesis as well as increased epigenetic age in high mtDNA deletion mutation iPSC, consistent with changes occurring in cellular aging. Together, these data demonstrate that high mtDNA mutation heteroplasmy induces changes occurring in cellular aging.

## Graphical abstract

We reprogrammed three fibroblast lines with heteroplasmic deleterious mitochondrial genome mutations to generate 76 induced pluripotent stem cell clones. Quantification of heteroplasmy in resultant clones demonstrates a bimodal segregation of heteroplasmy levels in induced pluripotent stem cells. Functional analysis reveals altered growth dynamics and regulation of metabolism in induced pluripotent stem cell clones with high levels of mitochondrial genome mutation heteroplasmy as compared to clones with the same nuclear genome but absent mitochondrial mutation heteroplasmy.

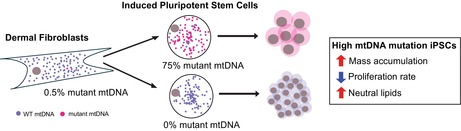

## 1 INTRODUCTION

Mitochondria are the “power plants” of eukaryotic cells, occurring in hundreds to thousands of copies per cell and generating up to 90% of cellular ATP (Harris & Das, 1991). In addition to energy generation, they have a central role in regulating cell behavior and identity through titration of cellular metabolites (Chakrabarty & Chandel, 2021). Each human mitochondrion contains multiple copies of a 16,569 base pair circular genome that transcribes *mRNAs*, *rRNAs*, and *tRNAs* for translating encoded proteins essential for respiration and ATP production. The mitochondrial genome (mtDNA) is mutated at a ~ 10‐to‐100‐fold higher rate than the nuclear genome (Allio et al., 2017; Ludwig et al., 2019). Due to the compact, intron‐less nature of mtDNA, mutations or deletions of any size could have a large impact on cellular metabolism. When a variant arises in mtDNA it can occur in all copies (“homoplasmy”) or in a fraction of the copies (“heteroplasmy”). The proportion of mutant mtDNA is referred to as the level of heteroplasmy. The impact of a mutation on cell function varies with the level of heteroplasmy and the cell type in which it occurs (Picard et al., 2014).

Alterations in mtDNA have been frequently observed in aged tissues and multiple disease states. Whereas low levels of pathogenic heteroplasmy are observed without apparent consequence in healthy individuals, high levels of heteroplasmy correlate with tissue dysfunction (Baris et al., 2015; Bua et al., 2006; Smith et al., 2020). Single cell studies suggest that high‐level heteroplasmy occurs through preferential clonal amplification of deleterious variants in specific cell types (Cao et al., 2001; Khrapko et al., 1999; Taylor et al., 2003), although the processes by which this occurs remain unclear. Once present, cellular functional consequences for high‐level mtDNA heteroplasmy are not well understood. Other types of mitochondrial dysfunction such as inhibition of the electron transport chain or depletion of key mitochondrial enzymes, can trigger age‐associated phenotypes of decreased proliferation, stem cell differentiation, cellular senescence or cell death (Bahat & Gross, 2019; Cheema et al., 2015; Kaplon et al., 2013; Mandal et al., 2011; Park et al., 2010; Wiley et al., 2016). Yet, it remains unknown whether clonal amplification of deleterious heteroplasmic mtDNA mutations are sufficient to trigger these changes, and therefore the functional relevance of mtDNA changes observed with in vivo aging remains open to debate.

The consequences of heteroplasmic mutations have been challenging to study as genome editing for mtDNA is limited and there are few established tools to alter heteroplasmy in vitro. Model systems such as the “mtDNA mutator” mouse containing a mutant polymerase gamma implicate mtDNA changes in many aging phenotypes (Trifunovic et al., 2004). However, this mouse model induces a large mix of genome alterations often with mtDNA depletion in cells, yielding much more disruption than the clonally expanded heteroplasmic mutation events that occur in usual aging in vivo. Much of our current knowledge regarding heteroplasmy comes from comparisons of primary cells from patients with mtDNA mutations to controls, often with low mutant heteroplasmy and unmatched nuclear genetics (Majora et al., 2009), or from immortal “cybrid” cells (Picard et al., 2014), which have a malignant pathophysiology and limit the capacity to study the impact of heteroplasmy on cell fate and viability.

Reprogramming somatic cells to pluripotency has been shown to reverse some markers of aging (Horvath, 2013; Miller et al., 2013), and expression of reprogramming factors is proposed as a potential rejuvenating therapy (Sarkar et al., 2020). However, the impact of mtDNA heteroplasmy on this process has not been queried. Although heteroplasmy of pathogenic mtDNA variants is typically stable for differentiated cells in culture, multiple recent studies established that heteroplasmy shifts significantly with reprogramming of primary cells to induced pluripotent stem cells (iPSCs). Many patterns of heteroplasmy shift have been noted, including large increases or total clearance of pathogenic mtDNA variants (Sercel et al., 2021; Wei et al., 2021). Prior studies established that iPSCs with high‐level mtDNA heteroplasmy show decreased mitochondrial respiration and maintain pluripotency (Chichagova et al., 2017; Ma et al., 2015; Russell et al., 2018; Yokota et al., 2015). However, beyond this single‐measure characterization, the impact of altered heteroplasmy on cell function, and particularly on the capacity for rejuvenation remains unexplored. This is a key area to understand as critical roles are rapidly evolving for mitochondrial metabolism in both maintenance of pluripotency and stem cell differentiation (Chakrabarty & Chandel, 2021).

We note that the differential segregation of mtDNA heteroplasmy following iPSC generation offers a novel opportunity to understand the impact of clonal increases or decreases in mtDNA heteroplasmy on cellular function in iso‐nuclear genome comparisons. We hypothesize that iPSCs with increased mtDNA heteroplasmy have functional adaptations consistent with cellular aging. Thus, we generated iPSC colonies from three primary fibroblast lines with known heteroplasmy of deleterious mtDNA mutations and quantified heteroplasmy of these mutations in resultant clones. We report that resultant clones displayed a primary bimodal distribution of mutation heteroplasmy. We determined that high‐level mtDNA deletion mutant iPSCs exhibit distinct growth properties, metabolic profiles, and altered differentiation capacity, with growth and metabolic shifts mirroring a key subset of changes observed in aging‐induced cell and tissue dysfunction.

## 2 RESULTS

### 2.1 Bimodal shifts in mtDNA mutation heteroplasmy with reprogramming to pluripotency

In order to quantify the impact of reprogramming on mtDNA heteroplasmy, we reprogrammed three non‐immortalized primary dermal fibroblast lines containing known pathogenic heteroplasmic mtDNA variants. Prior to reprogramming, we performed an initial characterization to validate the presence of mutations and assess baseline mitochondrial respiration. One line contained mtDNA with heteroplasmy for a commonly studied point mutation affecting the leucine *tRNA*, A3243G, and two lines contained mtDNA with heteroplasmy of the most frequently identified mtDNA deletion, Δ4977 (Majora et al., 2009). For cells containing the A3243G point mutation, the presence of the mutation was validated using Sanger sequencing (Figure S1a), and the mutation was quantified at 89% using ARMS‐qPCR (Bai & Wong, 2004). The presence of the Δ4977 deletion was validated using targeted nanopore sequencing (Vandiver et al., 2023) and quantified using droplet digital PCR with primers specific to the known deletion (Pogozelski et al., 2003). One line had 1.49% of Δ4977 mtDNA, whereas the second line contained 0.45% of Δ4977 mtDNA (Figure S1b,c). We measured baseline respiration with a Seahorse Extracellular Flux assay (Figure S1d). This assessment showed reduced basal and maximal respiration of A3243G heteroplasmic fibroblasts compared to a control neonatal dermal fibroblast, which were derived from a different subject. By contrast, neither Δ4977 heteroplasmic fibroblast line showed reduced respiration compared to the control, consistent with inconsequential low‐level heteroplasmy.

**FIGURE 1:**
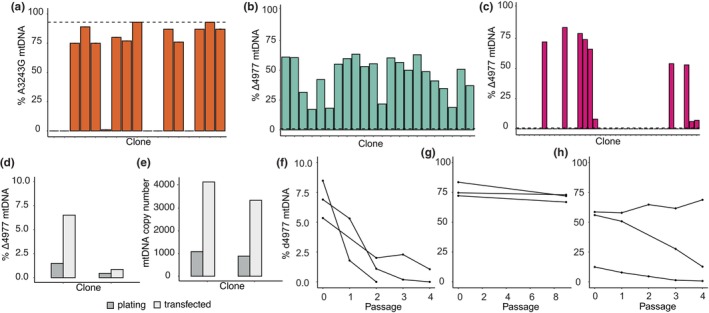
Mitochondrial DNA heteroplasmy shifts dynamically with reprogramming to pluripotency. (a) ARMS‐qPCR quantification of A3243G heteroplasmy in passage 0 iPSC clones. Dashed line indicates heteroplasmy level of parental fibroblast line. (b, c) DdPCR quantification of Δ4977 heteroplasmy in passage 0 iPSC clones. Dashed line indicates heteroplasmy level of parental fibroblast lines. (d, e) DdPCR quantification of Δ4977 heteroplasmy (d) and mtDNA copy number (e) in two parental fibroblast lines prior to reprogramming (dark grey) and 1 week after Sendai virus transfection (light grey). (f–h) DdPCR quantification of Δ4977 heteroplasmy in three low‐level heteroplasmy iPSC clones (f), three high‐level heteroplasmy iPSC clones (g) and three intermediate‐level heteroplasmy iPSC clones (h) with serial passage.

Following this initial characterization, the three dermal fibroblast lines were reprogrammed to iPSCs using Sendai virus expression of *OCT4*, *KLF4*, *SOX2* and *c‐MYC* (Fusaki et al., 2009). A total of 76 individual, independent iPSC clones were generated, including 18 clones from A3243G heteroplasmic dermal fibroblasts, 22 clones from the 1.49% Δ4977 heteroplasmic fibroblasts and 36 clones from the 0.45% Δ4977 heteroplasmic fibroblasts. We assessed the mtDNA mutation heteroplasmy of passage 0 iPSC clones using ARMS‐qPCR for A3243G clones and ddPCR for Δ4977. Quantification of heteroplasmy in these clones revealed an intriguing pattern. For iPSCs derived from high‐level A3243G mutant fibroblasts, clones either maintained nearly parental level heteroplasmy or had no evidence of point mutant heteroplasmy (Figure 1a). For iPSCs derived from Δ4977 heteroplasmic fibroblasts, we measured either a large amplification of the deletion mutation heteroplasmy or clearance of the deletion (Figure 1b,c). To validate ddPCR quantitation of heteroplasmy using an orthogonal method, targeted nanopore sequencing (Vandiver et al., 2023) was performed on one high‐level mutant Δ4977 iPSC clone and one WT Δ4977 iPSC clone. These data confirmed that the specific deletion event detected in the parental fibroblast line was present in 78% of the reads in the high‐level mutant clone and no reads were present in the no‐mutant, WT reversion clone, consistent with ddPCR quantification (Figure S2).

Because of the large shift in mtDNA heteroplasmy noted with reprogramming of Δ4977 heteroplasmic fibroblasts, we next sought to quantify the pattern of heteroplasmy shift. We first used ddPCR to examine mtDNA isolated from parental fibroblasts at 1 week following Sendai virus transfection, a time point when the cells had not yet taken on a stem cell morphology. At this time point, a four‐fold and a two‐fold increase in Δ4977 heteroplasmy were observed (Figure 1d), coupled with an over four‐fold increase in mtDNA copy number (Figure 1e), indicating that while mtDNA was being globally amplified, there was also a preferential increase in Δ4977 genomes.

We next determined whether distinct patterns of heteroplasmy maintenance could explain the bimodal distribution of mtDNA heteroplasmy obtained at endpoint by examining three clones with <10% of Δ4977 mtDNA at passage 0 (P0), three clones with high‐level Δ4977 heteroplasmy at P0, and three clones with intermediate level heteroplasmy of Δ4977 mtDNA at P0. We expanded each clone in culture and quantified heteroplasmy at each passage using ddPCR. Heteroplasmy rapidly declined with expansion of cells with <10% Δ4977 mtDNA, with no detectable Δ4977 mtDNA in all but one clone by passage 4 (Figure 1f). By contrast, expansion of three high‐level Δ4977 mtDNA clones showed persistent high‐level heteroplasmy up to passage 9 (Figure 1g). Meanwhile, clones from the second set of Δ4977 iPSCs that initially had intermediate level heteroplasmy at P0 moved towards the low or high extremes by the fourth passage (Figure 1h).

### 2.2 iPSCs with high‐level mtDNA mutant heteroplasmy undergo trilineage differentiation

**FIGURE 2:**
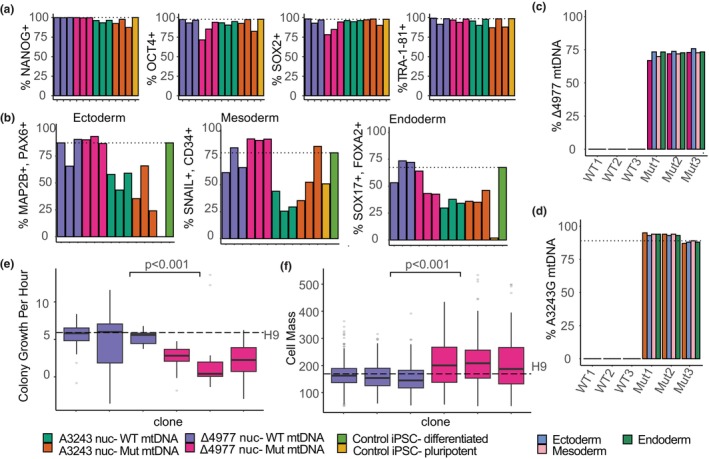
IPSCs with high‐level mtDNA mutation heteroplasmy undergo trilineage differentiation. (a) Percentage of iPSCs expressing pluripotency markers NANOG, OCT4, SOX2 and TRA‐1‐81 as quantified by flow cytometry. (b) Percentage of putative lineage progenitor cells derived from each iPSC clone expressing dual lineage biomarkers. (c) DdPCR quantification of Δ4977 heteroplasmy in iPSCs (dark pink) and putative lineage progenitors derived from each iPSC clone (light blue denotes ectoderm, light pink denotes mesoderm, light green denotes endoderm). (d) ARMS‐qPCR quantification of A3243G heteroplasmy in iPSCs (dark orange) and putative lineage progenitors derived from each iPSC clone (light blue denotes ectoderm, light pink denotes mesoderm, light green denotes endoderm). (e) iPSC clone colony biomass accumulation rates percent per hour quantified by live cell interferometry (*n* = 53, *p* < 0.001). (f) iPSC single cell absolute biomass in picograms quantified by live cell interferometry (*n* = 863, *p* < 0.001).

We next used the established iPSC clones to query whether high‐level mtDNA heteroplasmy affects maintenance of a pluripotent identity. We expanded three iPSC clones with high‐level A3243G heteroplasmy generated from A3243G fibroblasts and three clones without A3243G heteroplasmy generated from A3243G fibroblasts as a control for the A3243G nuclear background. In addition, three clones with high‐level Δ4977 heteroplasmy generated from Δ4977 fibroblasts and three clones without Δ4977 heteroplasmy generated from Δ4977 fibroblasts were expanded as a control for the Δ4977 nuclear background in culture. At passage 7, we assessed all clones for Sendai virus expression and obtained no evidence of persistent expression (Figure S3a). We screened all clones for common PSC genetic aberrations and obtained no evidence of aneuploidies (Figure S3b). We then evaluated expression of pluripotency markers, with all clones showing high expression of OCT4, SOX2, NANOG, and TRA‐1‐81 by flow cytometry (Figure 2a).

To assess the capacity for trilineage differentiation characteristic of pluripotency, we performed lineage‐directed differentiation and measured the induction of ectoderm, mesoderm, and endoderm lineage‐specific biomarkers following stimulation of each clone. Although there was variation between clones in their levels of differentiated biomarker expression, each clone acquired appropriate germ lineage differentiation biomarkers (Figure 2b) with attendant loss of pluripotency biomarkers (Figure S4), indicating that the capacity for differentiation was unaltered even with high‐level mtDNA mutant heteroplasmy. Quantification of mtDNA heteroplasmy following germ lineage directed differentiation indicated stable heteroplasmy for both A3243G and Δ4977 clones in each differentiated lineage (Figure 2c,d), further supporting the stability of high‐level heteroplasmy in iPSC clones and their progeny.

### 2.3 iPSCs with high‐level mutation heteroplasmy have altered growth and differentiation

Although all expanded iPSC clones met criteria for pluripotency, slower growth was noted for clones with high‐level heteroplasmy of the Δ4977 mutation, which required less frequent passaging than clones derived from the same Δ4977 fibroblasts without Δ4977 mtDNA. In order to quantify growth differences, iPSC clones with and without high‐level mtDNA deletion heteroplasmy were analyzed using live cell interferometry, a quantitative phase microscopy technique adapted for living cells, which was previously optimized for pluripotent stem cell growth analyses (Zangle et al., 2013). Growth quantification showed significantly slower iPSC colony biomass accumulation in clones with high‐level Δ4977 heteroplasmy compared to colonies derived from the same parental fibroblasts but without Δ4977 mtDNA (Figure 2e). In addition, individual cells of the high‐level Δ4977 clones had significantly more biomass than clones lacking Δ4977 mtDNA (Figure 2f), indicating that high‐level Δ4977 iPSC clones accumulated more biomass prior to triggering cell division.

**FIGURE 3:**
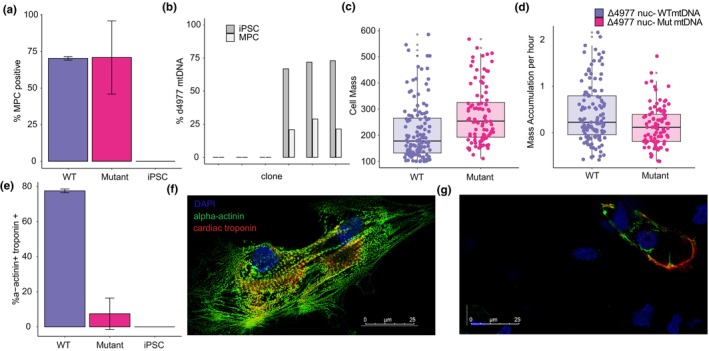
High‐level mutant mtDNA iPSC clones alter the phenotype of differentiated cells. (a) Percentage of cells expressing MPC biomarkers CD90, CD105, CD73 and negative for expression of CD45, CD11b, CD19, HLA‐DR and SOX2 biomarkers, as quantified by flow cytometry (*n* = 3, *p* = 0.97). (b) DdPCR quantification of Δ4977 heteroplasmy in iPSC clones (dark grey) and MPC lines derived from each iPSC clone (light grey). (c) MPC cell biomass in picograms measured by live cell interferometry (*n* = 92, *p* < 0.01). (d) MPC percent growth per hour measured by live cell interferometry (*n* = 92, *p* < 0.01). (e) Percentage of cells expressing cardiomyocyte biomarkers alpha‐actinin and cardiac troponin (*n* = 2, *p* = 0.05). (f, g) Immunocytochemistry visualization of cardiomyocyte biomarkers alpha‐actinin (green), cardiac troponin (red) in putative cardiomyocytes derived from WT iPSC clones (f) and iPSC clones with high‐level Δ4977 heteroplasmy (g).

Because of the growth differences observed, we also wondered whether high‐level mtDNA mutations affect the capacity for iPSC terminal differentiation. We first tested whether high‐level mutation heteroplasmy alters differentiation to mesenchymal progenitor cells (MPCs), a widely used progenitor cell type. Three high‐level Δ4977 mutant iPSC clones, and three nuclear genome matched WT mtDNA iPSC clones, were induced to differentiate into MPCs following a well‐established 21 day protocol (Patananan et al., 2020). At the end of differentiation, all clones had established iPSC‐derived MPCs with typical spindle cell morphology (Figure S5a,b). Flow cytometry analyses demonstrated similar levels of cells meeting ISCT biomarker criteria for MPC identity (Dominici et al., 2006) for both high‐level mutant mtDNA and WT mtDNA clones (Figure 3a), indicating unaltered capacity for MPC differentiation in high‐level mutant iPSC clones. However, quantification of heteroplasmy following differentiation indicated a significant (*p* < 0.01) decrease in deletion heteroplasmy following successful MPC differentiation (Figure 3b). Quantification of mtDNA copy number demonstrated variability between clones, but no significant difference in mtDNA copy number corresponding to Δ4977 mutation heteroplasmy (Figure 5c). Despite this reduced heteroplasmy, similar patterns of growth to that observed in the iPSC state were observed, with high‐level mutant mtDNA MPCs showing significantly higher per cell biomass (Figure 3c) and significantly slower growth (Figure 3d) compared to MPCs differentiated from WT mtDNA iPSC clones. To determine whether this altered growth phenotype impacted the capacity for terminal differentiation, MPCs were induced to differentiate into adipocytes and osteoblasts following another well‐established 21 day protocol (Patananan et al., 2020), which showed an intact capacity for both adipogenesis and osteogenesis in both high‐level mutant mtDNA and WT mtDNA clones (Figure S5d).

Although high‐level mutant iPSC clones were capable of MPC differentiation, we speculated that limitations in differentiation may arise in cell types with higher dependency on aerobic respiration than MPCs. To evaluate this prospect, we attempted directed differentiation to ventricular cardiomyocytes following an established 14‐day differentiation protocol (Ye et al., 2020). Although all iPSC clones produced viable cells at the end of the directed differentiation period, coordinated beating was only observed in WT mtDNA cells, not in cells differentiated from high‐level Δ4977 mtDNA iPSC clones (Figure S5e). Furthermore, expression of ventricular cardiomyocyte biomarkers alpha‐actinin and cardiac troponin was significantly reduced in high‐level Δ4977 mtDNA cells compared to WT mtDNA cells (Figure 3e–g), indicating a reduction of differentiation capacity to functional cardiomyocytes in the presence of high‐level Δ4977 mtDNA. As observed with MPCs, quantification of mtDNA copy number demonstrated similar levels between mtDNA genotypes (Figure S5f).

### 2.4 High‐level mtDNA deletion mutation iPSCs have an altered transcriptome

**FIGURE 4:**
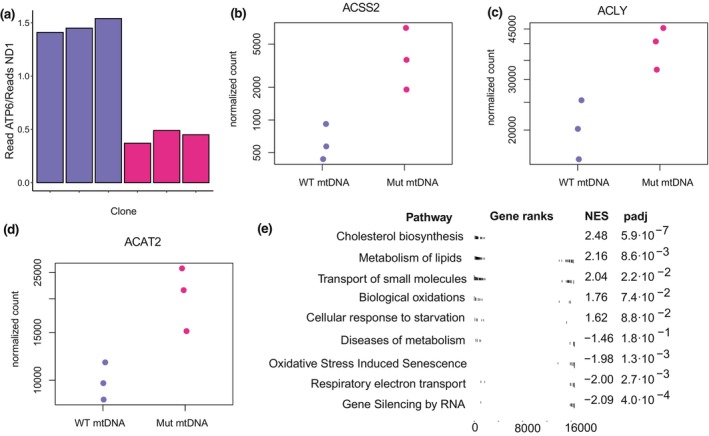
Transcriptional alterations support a metabolic shift in iPSC clones with high‐level mtDNA mutation heteroplasmy. (a) Ratio of transcripts of *ATP6*, a gene within the deletion region of Δ4977 mtDNA, to *ND1*, a gene not within the deletion region, for iPSC clones containing WT mtDNA (shown in blue) and Δ4977 heteroplasmic mtDNA (shown in dark pink). (b–d) Normalized transcript count for *ACSS2*, *ACLY* and *ACAT2* in iPSC colonies. (e) Gene set enrichment analysis of genes differentially expressed between high‐level Δ4977 mtDNA and WT mtDNA iPSC clones. Shown are condensed pathways including more than 10 genes.

With a significant growth difference in the context of high‐level mtDNA deletion heteroplasmy, we sought to determine which pathways may underlie this altered growth phenotype. We first investigated the impact of mtDNA heteroplasmy on the pluripotent cell transcriptome. We performed RNA‐sequencing on RNA isolated from passage 9 clones of three high‐level Δ4977 mutant iPSC clones and three iPSC clones from the same parental fibroblasts without Δ4977 mtDNA. An average of 78,250,000 reads per sample were generated with an average of 80.53% of reads mapping to the human transcriptome. Analysis of the mitochondrial transcriptome supported significant impact of mtDNA heteroplasmy on expression of the electron transport chain proteins encoded in the deleted region, with expression of genes within the deletion relative to unaffected genes proportionate to the 70–75% heteroplasmy quantified by ddPCR (Figure 4a). Analysis of differentially expressed genes identified 234 genes significantly differentially expressed in association with high‐level Δ4977 mutation (Table S1). These included two key regulators of cytoplasmic acetyl‐CoA levels, *ACSS2* (*p* adjusted<0.01) and *ACLY* (*p* adjusted <0.01), as well as the regulator of cytoplasmic acetyl‐CoA usage, *ACAT2* (*p* adjusted <0.01) (Figure 4b–d). Gene set enrichment analysis supported a significant change in the expression of genes involved in regulating the cellular metabolome and response to stressors. Increased expression of genes associated with cholesterol and lipid metabolism were detected, as was enrichment for pathways involved in the response to oxidative stress and transcription regulation (Figure 4e).

### 2.5 High‐level mtDNA mutant iPSC clones exhibit metabolic reprogramming

Because of the strong transcriptional signal for metabolic change, we investigated the specific impact of high‐level mtDNA mutant heteroplasmy on iPSC metabolism. To assess mitochondrial function in each set of iPSC clones, each clone was analyzed using the Seahorse Extracellular Flux Analyzer. Results showed a decreased basal oxygen consumption rate and decreased percentage of ATP produced by oxidative phosphorylation in clones containing high‐level Δ4977 mtDNA heteroplasmy compared to clones derived from the same fibroblasts without Δ4977 heteroplasmy (*p* < 0.01, *p* = 0.07). A similar result was also obtained in comparison to iPSC clones containing high‐level A3243G heteroplasmy compared to clones derived from the fibroblasts without A3243G heteroplasmy (*p* < 0.01, *p* < 0.01), with high‐level Δ4977 iPSC clones exhibiting more extreme reductions than high‐level A3243G iPSC clones (Figure S6).

**FIGURE 5:**
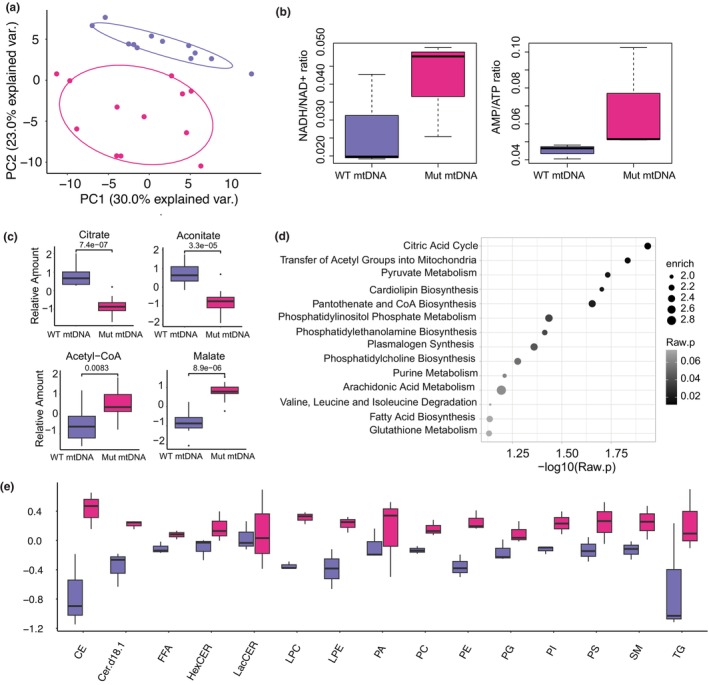
Metabolic shifts in high‐level Δ4977 mtDNA mutant iPSC clones. (a) Principal component analysis of 122 normalized metabolites measured by UHPLC–MS in iPSC clones containing WT mtDNA (purple) and high‐level Δ4977 mtDNA (dark pink). (b) NADH/NAD+ and AMP/ATP ratios from normalized metabolic data for iPSC clones containing WT mtDNA (purple) and high‐level Δ4977 mtDNA (dark pink). (c) Normalized TCA cycle metabolites from iPSC clones containing WT mtDNA (purple) and high‐level Δ4977 mtDNA (dark pink). (d) Metabolite set enrichment analysis for differentially present metabolites between iPSC clones containing WT mtDNA and high‐level Δ4977 mtDNA. (e) Normalized mean lipid levels for 13 classes of lipid species profiled by shotgun lipidomics.

To understand the impact of reduced mitochondrial function on the pluripotent metabolome, we analyzed the three high‐level Δ4977 mtDNA iPSC clones and the three matched non‐mutant mtDNA iPSC clones using steady state metabolomics. A set of 122 metabolites were identified by ultra‐high‐performance liquid chromatography mass spectrometry (Table S2). Unsupervised clustering demonstrated distinct metabolic profiles between high‐level Δ4977 mtDNA clones and matched controls (Figure 5a). Analysis of energetic intermediates demonstrated a non‐significant trend towards increased AMP to ATP and NADH to NAD+ ratios, as also reported in other models of mitochondrial dysfunction and associated with cell senescence (Figure 5b) (Khaidizar et al., 2021; Wang et al., 2003). Surprisingly, many TCA cycle intermediates, including the established pluripotency regulator alpha‐ketoglutarate, were not differentially present. However, citrate and aconitate were reduced in the high‐level Δ4977 mtDNA mutant iPSC clones, whereas malate and acetyl‐CoA were elevated in these mutant clones (Figure 5c), suggesting a shift in metabolite abundance that is potentially consistent with the export of acetyl‐CoA to the cytosol. In addition to TCA cycle metabolites, enrichment for other pathways involving regulation of acetyl‐CoA, including pyruvate and fatty acid metabolism, was detected amongst differentially abundant metabolites (Figure 5d).

Given the indication of altered lipid synthesis in both RNA sequencing and metabolomic data, we performed shotgun lipidomics to profile 13 lipid classes in three iPSC clones with high‐level Δ4977 mtDNA and matched clones without the mutation (Table S3). Intriguingly, this comparison profiling showed a broad increase across all classes of lipids for high‐level Δ4977 mtDNA mutant clones, with the most prominent increases in triacylglycerols and cholesterol (Figure 5e), two neutral lipids that are stored in lipid droplets and are known to increase in multiple models of senescence and aging (Bresgen et al., 2023; Lizardo et al., 2017).

### 2.6 High‐level deletion mutant iPSCs show altered epigenetic age

**FIGURE 6:**
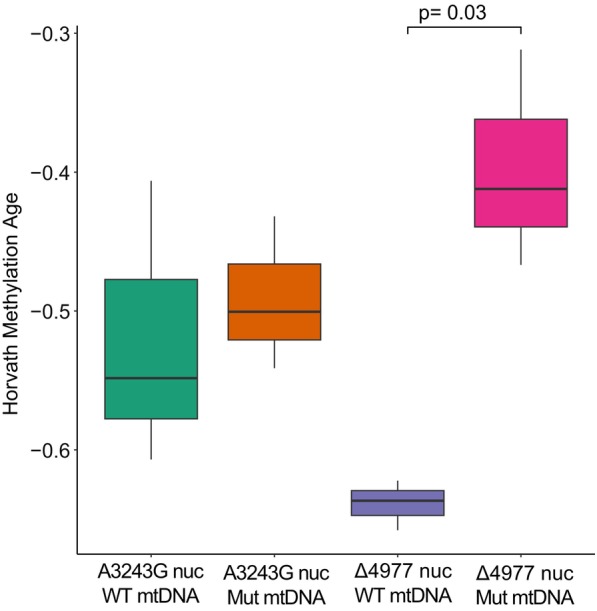
DNA methylation age of iPSC is increased in high deletion mutation clones. Shown is DNA methylation age calculated using the Horvath, 2013 formula for iPSC clones with absent or high‐level A3243G point mutation or Δ4977 deletion mutation.

The observed differences in cell growth dynamics and lipid composition led us to question whether other changes associated with cellular aging might be observed in iPSC containing high‐level mtDNA deletion heteroplasmy. DNA methylation age is a well‐established marker of tissue aging that increases with cell passage and reverts to below zero with induction of pluripotency (Horvath, 2013). Low methylation ages are necessary for pluripotency, however, an increase in this age has been correlated with donor age even within iPSCs (Lo Sardo et al., 2017). To determine the impact of high‐level mtDNA mutation heteroplasmy on epigenetic age reversal, genome wide methylation was determined from parental fibroblasts and iPSC clones at passage 9 using the Infinium Epic Array and methylation age was calculated using the original Horvath methylation clock (Horvath, 2013). Although all iPSCs showed reversion of methylation age from parental fibroblasts (Figure S7), iPSC methylation age varied with mtDNA mutation level and respiratory function. Within iPSCs derived from A3243G fibroblasts, iPSCs with high‐level mutation, which displayed a low degree of change in respiration and growth, showed a non‐significant increased epigenetic age (Welch *t*‐test, *p* = 0.69). In contrast, iPSCs derived from Δ4977 fibroblasts and high‐level deletion mutation iPSCs show significant changes in respiration and growth and demonstrate a significant increase in DNA methylation age (Figure 6) (Welch *t*‐test, *p* = 0.02). Although the magnitude of change in methylation age is low compared to differences reported for somatic cells (Horvath, 2013), the level of variation observed between WT mtDNA and high‐level deletion mutant mtDNA iPSCs is greater than that observed between iPSCs derived from 20‐year‐old donor cells compared to iPSCs derived from 100‐year‐old donor cells (Lo Sardo et al., 2017).

## 3 DISCUSSION

Changes in mitochondrial mutation heteroplasmy occur frequently in aging tissue, but the functional consequences of these shifts remain poorly understood due to a lack of systems to examine this issue. Reprogramming cells to pluripotency induces large shifts in mtDNA heteroplasmy (Sercel et al., 2021; Wei et al., 2021), offering an opportunity to understand changes in cell behavior brought about by altered heteroplasmy. We queried the trajectory and consequences of shifts in heteroplasmy by reprogramming fibroblasts with heteroplasmy of two frequently observed deleterious mtDNA mutations. We observed a bimodal pattern of heteroplasmy shift, with either high‐level heteroplasmy or clearance of heteroplasmy for both mutations studied. We identified significant functional consequences of elevated clonal deletion heteroplasmy in iPSCs and in differentiated progeny cells. iPSC clones with high‐level heteroplasmy of a common mtDNA deletion showed altered growth kinetics, increased neutral lipid synthesis and increased epigenetic age, similar to changes that occur with stem cell aging in vivo and cell senescence in vitro (Bresgen et al., 2023; Khaidizar et al., 2021; Lizardo et al., 2017; Marschallinger et al., 2020; Wang et al., 2003).

An intriguing study result is the occurrence of a bimodal distribution pattern of heteroplasmy following reprogramming. Prior work suggested that such a pattern could be evidence of heterogeneity of the initial cell population (Sercel et al., 2021). However, our detection of a more varied distribution of heteroplasmy at passage 0 with segregation to a bimodal heteroplasmy pattern by passage 4 suggests an active process enabling either expansion or complete clearance of deleterious heteroplasmic variants. The marked expansion of mtDNA containing deletions is similar to the preferential amplification of mtDNA containing deletions observed in other systems following ethidium bromide depletion of mtDNA or induction of DNA double‐stranded breaks (Diaz et al., 2002; Fukui & Moraes, 2009). Our results differ from another study that showed only expansion of a different deletion mutant with reprogramming (Russell et al., 2018), although that study considered a much smaller number of iPSC clones, so it is possible that not enough clones were profiled to detect the bimodal pattern. Our findings are distinct from but potentially supportive of recent work analyzing mtDNA heteroplasmy in iPSCs using a single cell approach (Wei et al., 2021), in which many fibroblast mtDNA mutations were cleared whereas others increased with reprogramming. In that study, the variants examined were at low‐level heteroplasmy and not identified as functionally relevant in the parental fibroblasts, making it possible that a different process regulates heteroplasmy for high‐level deleterious mutations. Our finding that low levels of heteroplasmy were cleared by passage of iPSC clones in culture supports prior work demonstrating potential selection against low levels of deleterious mtDNA mutations in prolonged iPSC culture (Kosanke et al., 2021). A molecular mechanism for bimodal segregation of mtDNA heteroplasmy remains unknown. In studies of other models of mtDNA disease, downregulation of autophagy occurs in cells with high‐level heteroplasmy (Bhattacharya et al., 2022). We suggest it is possible that a similar process occurs for iPSC clones that reach a heteroplasmy threshold, allowing maintenance of elevated heteroplasmy, but not for those with lower level heteroplasmy for which mitophagy then clears deleterious mutations.

The observed bimodal segregation of mutation heteroplasmy with reprogramming reveals the dynamics of mtDNA heteroplasmy regulation in a permissive setting in replicating cells, although it is unknown whether these patterns translate to human tissues. In humans, there is significant evidence of tissue specific shifts in mtDNA point mutation heteroplasmy, including clearance of mutations in blood (Rahman et al., 2001). This specificity may relate to the metabolic requirements of each tissue type, as suggested with our observed reduction of heteroplasmy with MPC differentiation. Furthermore, in human tissues, mtDNA deletion mutations accumulate at high levels in non‐dividing tissues (Bua et al., 2006; Kraytsberg et al., 2006) rather than the rapidly proliferative cells in our model. We note that mtDNA replication is persistent in post‐mitotic cells (Magnusson et al., 2003), so the observed replicative advantage may still apply, as occurs in mouse neurons (Fukui & Moraes, 2009; Lawless et al., 2020), although our current work examines these heteroplasmy dynamics only in iPSCs.

The ability of high deletion mutation iPSCs to generate functional MPCs, osteoblasts, and adipocytes, but not cardiomyocytes, is intriguing. These findings uncover the importance of mtDNA heteroplasmy in the relationship between mitochondrial metabolism and terminal cell differentiation. However, it remains unknown how this occurrence translates into clinical mtDNA mutation syndromes, in which levels of mutation heteroplasmy vary with stages of development and tissue type (Rahman et al., 2001). Children born with mtDNA deletion linked syndromes do not have a deficit in heart formation; however, the levels of mutant mtDNA heteroplasmy in embryonic cardiac tissue from these individuals has not been queried. Cardiac abnormalities have been reported throughout life for some individuals with mtDNA deletions (Jennifer & Cortez, 2020; Trivedi et al., 2018). Prior work demonstrated that mtDNA depletion is associated with abnormalities of heart formation (Larsson et al., 1998), which suggests that high mtDNA deletion iPSCs could fail to establish mature cardiomyocytes. Both media acidification and glucose induced ROS production have been established to increase cardiomyocyte production in other settings (Crespo et al., 2010; Liu et al., 2024), reinforcing a central role for metabolism in heart cell differentiation. Both processes are also intimately linked to mitochondrial function, suggesting that future work examining the impact of mtDNA heteroplasmy on these parameters may overcome the identified differentiation barrier.

A novel finding in our work is the potential impact of mtDNA mutations on iPSC mass accumulation, growth rate, and cell division. As reported in other studies (Chichagova et al., 2017; Folmes et al., 2013; Fujikura et al., 2012; Grace et al., 2019; Ma et al., 2015; Prigione et al., 2011; Russell et al., 2018; Yokota et al., 2015), high‐level mtDNA mutant iPSCs maintain pluripotency and show skewed differentiation potential (Meshrkey et al., 2023). We additionally observe distinct growth patterns with increased mass accumulation prior to cell division. This pattern is reminiscent of changes observed in murine iPSCs carrying a mutator phenotype in the mitochondrial polymerase‐γ (Hämäläinen et al., 2015). However, our work shows this growth deficit in human iPSCs with only mutation heteroplasmy, and without pleotropic effects on mitochondrial replication caused by polymerase mutants. Furthermore, we report this same growth pattern in MPCs differentiated from iPSC clones persists, extending relevance to differentiated progenitor cells. While high mtDNA deletion mutant cells remain proliferative, they shift to increasing mass accumulation before division, a pattern that occurs in both cellular senescence and stem cell aging (Lengefeld et al., 2021; Ogrodnik, 2021). We also detect metabolic shifts with a reduction in relative NAD+ and ATP levels and an increase in neutral lipids, resembling changes in some studies of cellular senescence (Khaidizar et al., 2021; Lizardo et al., 2017; Wang et al., 2003) and directly linked to tissue dysfunction in disease (Bresgen et al., 2023; Conte et al., 2013; Marschallinger et al., 2020). Mitochondrial dysfunction independent of mtDNA genotype has similar metabolic shifts and induction of cell senescence (Bahat & Gross, 2019; Kaplon et al., 2013; Mandal et al., 2011; Park et al., 2010; Wiley et al., 2016), it nevertheless remains controversial whether increased heteroplasmy of single mtDNA mutations would be sufficient to induce this change. Our data directly demonstrate that an increase in heteroplasmy of a single deleterious mutation is sufficient to establish aging‐related shifts in cell growth.

Our findings speak to recent interest in the relationship between reprogramming and cellular aging. Reprogramming reverses biomarkers of cellular aging (Miller et al., 2013; Horvath, 2013) and recent work shows that even transient expression of reprogramming factors has a rejuvenating benefit on cellular aging (Lu et al., 2020; Sarkar et al., 2020). However, the impact of mtDNA mutations on rejuvenation remains unexplored. MtDNA mutations are prevalent in aged human tissue, so understanding their impact on the capacity for reprogramming‐induced rejuvenation is essential before these approaches are considered in a human setting. Here, we show that deleterious mtDNA mutations can increase in heteroplasmy with reprogramming and significantly perturb cell growth kinetics, metabolism and differentiation capacity, all of which may limit the clinical potential of reprogramming for rejuvenation. Our study suggests that future work to understand reprogramming and transient reprogramming on mtDNA heteroplasmy will be essential before development of these approaches as anti‐aging therapies.

Limitations of this study include the use of two specific mtDNA mutations and on iPSCs for functional characterization of cells with high‐level mtDNA heteroplasmy. We picked the A3243G point mutation and Δ4977 deletion mutation because they are the most frequently reported deleterious mtDNA mutations, and both substantially increase in heteroplasmy in aging and disease (Grady et al., 2018; Yusoff et al., 2019). In future studies, it will be interesting to determine whether shifting patterns of heteroplasmy are similar for other known deleterious mtDNA deletion and point mutations. Our choice of using iPSCs enabled iso‐genic nuclei in a system for studying mtDNA mutations, which has not been possible in non‐immortalized cells. The relevance of the observed findings to other cell types, including tissue stem cells, gains support by the similar growth pattern measured in iPSCs and in iPSC‐derived MPCs, although disparate findings with cardiomyoctye differentiation indicates the cell type specific effects for mtDNA mutations that remain an important area for further investigation. Furthermore, this work utilizd iPSCs propagated on Matrigel, a standard matrix for iPSC culture (Lu et al., 2022) that may introduce more variability than xeno‐free matrices (Rivera et al., 2020). Our findings on the physiological impact of bimodal segregation of mtDNA mutation heteroplasmy uncovers the importance of continuing to monitor mtDNA mutation heteroplasmy with the constantly evolving methods of iPSC generation and culture.

In summary, by reprogramming fibroblasts with known heteroplasmy for deleterious mtDNA mutations, we report a bimodal shift in heteroplasmy associated with reprogramming and demonstrate that elevated heteroplasmy of a deleterious mutation is sufficient to slow growth and shift metabolic programs, even in highly proliferative iPSCs. These findings directly link clonal expansion of mtDNA mutation heteroplasmy to regulation of proliferation and cell size, which was not possible to show in other in vitro models of heteroplasmy with differing nuclear genomes. The work provides a foundation for dissecting mechanisms connecting altered heteroplasmy to aging phenotypes and for understanding cell type specific effects of deleterious mtDNA heteroplasmy. Clones with iso‐genic nuclei and diverse levels of heteroplasmy show metabolic changes, providing a controlled system for testing targeted metabolic therapies to mitigate the observed growth and differentiation phenotypes.

## 4 METHODS

### 4.1 Cell lines

De‐identified dermal fibroblast lines containing Δ4977 mtDNA were obtained as a gift from Dr. Jean Krutmann (Majora et al., 2009). De‐identified dermal fibroblasts containing A3243G mtDNA were obtained from the NAMDC repository. Control hiPS2 (Male; RRID:CVCL_B508) primed iPSCs were obtained through the UCLA BSCRC hPSC Core Bank (Jerome Zack, UCLA).

All work with human pluripotent stem cells (hPSCs) were approved by UCLA Embryonic Stem Cell Research Oversight (ESCRO) Committee under Protocol # 2007–003‐17.

### 4.2 Cell culture

Fibroblasts were grown at 37°C and 5% CO_2_ in complete media containing DMEM (Corning, Cat. # 10013CV) supplemented with 10% Fetal Bovine Serum (FBS, Hyclone, Cat. # SH30088.03HI0), penicillin–streptomycin (Corning, Cat. # 30‐002‐CI), GlutaMax (ThermoFisher, Cat. # 35050‐061), non‐essential amino acids (MEM NEAA, ThermoFisher, Cat. # 11–140‐050) and 50 ug/mL uridine (Sigma, Cat. # U3003). Antibiotics were removed from the media for a minimum of one passage prior to cell reprogramming.

iPSCs were grown on matrigel (Corning, Cat. # 356234) coated plates in mTeSR Plus media (StemCell Technologies, Cat. # 85850) according to the manufacturer's protocol. No antibiotics were used. Cells were tested for mycoplasma contamination approximately every two passages using MycoAlert® Mycoplasma Detection Kit (Lonza, Cat. # LT07‐318).

### 4.3 Reprogramming

Fibroblasts were reprogrammed to iPSC using Cytotune 2.0 iPS‐Sendai Reprogramming kit (Fisher, Cat. # A16517) according to the manufacturer's instructions. Briefly, dermal fibroblasts were plated in a single well of a 6 well plate. Two days after plating, cells were transduced with CytoTune 2.0 Sendai reprogramming vectors and incubated overnight. Media was changed every 2 days, then on Day 7 post‐transduction, cells were transferred to MEF‐feeder plates. and cultured in DMEM/F12 (Gibco, Cat. # 11320033) supplemented with 20% KnockOut Serum Replacement (Gibco, Cat. # 10828–028), 1% Glutamax (Gibco, Cat. # 35050–061), 1% NonEssential Amino Acids (Gibco, Cat. # 11140–050), and 0.1 mM 2‐mercaptoethanol (Gibco, Cat. # 21985–023). Media was changed daily. When iPSC colonies emerged, they were picked and transferred to either new MEF‐feeder plates or Matrigel plates for expansion and genotyping.

### 4.4 Oxygen consumption measurements

OCR was measured using a Seahorse XF96 Extracellular Flux Analyzer (Agilent). 5 × 105 cells per well were seeded onto a matrigel coated V3 96‐well plate (Agilent, Cat. # 101085‐004) and grown overnight before analysis. A mitochondrial stress test quantified OCR at basal respiration and after the sequential addition of mitochondrial inhibitors oligomycin, carbonyl cyanide‐p‐trifluoromethoxyphenylhydrazone (FCCP), and rotenone. ATP production was quantified using oxygen consumption and extracellular acidification rates based on established methods (Desousa et al., 2023). For each clone, five technical replicates were plated and analyzed.

### 4.5 DNA extraction

Total DNA was extracted from frozen cell pellets using Qiagen DNeasy kit (Cat. #69504) in accordance with manufacturer's protocol.

### 4.6 Nanopore sequencing

A custom guide RNA sequence targeting human mtDNA: ACCCCTACGCATTTATATAG was used in accordance with previously described methods (Vandiver et al., 2022). Pre‐complexed Alt‐R CRISPR‐Cas9 single guide RNA (IDT) was diluted to a concentration of 10 μM and combined with HiFi Cas9 Nuclease V3 (IDT, cat 1,081,060) in CutSmart Buffer (NEB, cat B7204). Cas9 cleavage and library preparation was performed in accordance with previously described methods (Gilpatrick et al., 2020). Approximately 3 μg of input DNA was dephosphorylated with alkaline phosphatase (ONT, cat SQK‐CS9109) in CutSmart Buffer (NEB, cat B7204). Following enzyme inactivation of alkaline phosphatase, 10 μL of 333 nM Cas9‐gRNA complex was combined with the dephosphorylated DNA, dATP (ONT, cat SQK‐CS9109), and Taq DNA polymerase (ONT, cat SQK‐CS9109). After Cas9 cleavage and dA‐tailing, sequencing adaptors were ligated to target DNA ends and library purification was performed using the Oxford Nanopore Technologies Cas9 Sequencing Kit (ONT, cat SQK‐CS9109). Samples were run on a MinION flow cell (v9.4.1) using the MinION Mk1C sequencer. Base calling was performed using Guppy (v4.5.4, release 4/20/21) on MinKnow. Base called reads with a qc score >5 were aligned to a custom mitochondrial genome reference sequence, rotated to extend from bp 1546 to bp 1545 of the modified cambridge chromosome M, using Minimap2 (Li, 2018), version 2.24. Deletions were then identified through parsing the CIGAR sequence of the primary alignment (Vandiver et al., 2022).

### 4.7 Droplet digital PCR for quantification of mtDNA copy number and Δ4977 deletion

For quantification of mtDNA copy number, droplet digital PCR was performed in accordance with previously reported methods (Herbst et al., 2021). Samples were diluted to the manufacturer's recommended target range (1 to 5000 copies per μL) in 25 μL reactions using the BioRad ddPCR Supermix for probes (BioRad, Cat.#1863024). Nuclear DNA was quantified using VIC‐conjugated primers targeting RNaseP (Fisher, Cat.#4401631), mtDNA was quantified using FAM‐conjugated primers targeting RNR1 (Fisher, Assay ID.#Hs02596859_g1)). The Δ4977 deletion was quantified using VIC‐conjugated deletion specific primers (Fwd CCTTACACTATTCCTCATCACC, Rev. TGTGGTCTTTGGAGTAGAAACC, Probe TGGCAGCCTAGCATTAGCAGT) (Pogozelski et al., 2003). Reactions were partitioned into droplets using a BioRad QX200 Droplet Generator (BioRad; Hercules, CA) prior to thermocycling. Digital PCR cycling conditions for nDNA and mtDNA copy number included Taq‐polymerase activation at 95°C for 10 min, followed by 40 cycles of denaturation at 94°C for 30 s and annealing/extension at 60°C for 2 min. Droplet fluorescence was then read using a BioRad QX200 Droplet Reader. Target copy numbers per microliter were determined using BioRad QuantaSoft Regulatory Edition Software (Version 1.7, BioRad; Hercules, CA).

### 4.8 Sanger sequencing for verification of A3243G mutation

A 1253 bp region was amplified from 100 to 200 ng of template DNA using Fwd primer cgagggttcagctgtctctt and gttcggttggtctctgcta Rev. Primer at 0.5 μM with 0.2 mM dNTPs 1X PCR buffer and 0.25 U of HotStar Taq (Qiagen Cat.# 203,205) with cycling conditions 95C × 15 min, 35 cycles of 95C × 0.5 min, 57C × 0.5 min, 72C × 1.5 min and a final extension at 72C for 10 min. Sanger sequencing was then run using the same primers (Laragen).

### 4.9 ARMS‐qPCR for quantification of A3243G heteroplasmy

Allele‐refractory PCR to quantify the A3243G point mutation was performed in accordance with previously published methods (Bai & Wong, 2004) after initial amplification of the target region. A 1253 bp region was amplified from 100 to 200 ng of template DNA using Fwd primer cgagggttcagctgtctctt and Rev. Primer ggttcggttggtctctgcta at 0.5 μM with 0.2 mM dNTPs 1X PCR buffer and 1.25 U of HotStar Taq (Quiagen Cat.# 203205) with cycling conditions 95C × 15 min, 35 cycles of 95C × 0.5 min, 57C × 0.5 min, 72C × 1.5 min and a final extension at 72C for 10 min. Amplicons were then diluted 1:40. For ARMS‐qPCR, 2 μL of each amplicon was used per reaction. The wildtype allele was amplified with FWD primer AGGGTTTGTTAAGATGGCTCA and REV primer TGGCCATGGGTATGTTGTTA, the A3243G allele was amplified with FWD primer AGGGTTTGTTAAGATGGCTCG and REV primer TGGCCATGGGTATGTTGTTA. All reactions were run with 0.5 μM of each primer, 1X SybrFast qPCR mastermix (Roche, Cat.# 07959494001) on a LightCycler480 (Roche). The percentage of mutant allele was calculated as 1/(1 + 1/2^(CT_WT‐CT_Mutant)^).

### 4.10 Intracellular flow cytometry

Cells were harvested using Gentle Cell Dissociation, then fixed in Fixation/Permeabilization solution (BD Biosciences, 554714) and incubated at 4°C for 30 m. Following fixation, cells were washed with 1x BD Perm/Wash Buffer (BD Biosciences, 554,714). Cells were re‐suspended in 100 μL of 1× BD Perm/Wash Buffer. Conjugated antibodies were incubated with fixed cells for 45 m at 4°C in the dark. Samples were processed on a LSRFortessa flow cytometer (BD Biosciences) and analyzed using FlowJo software (FlowJo, Inc.). The following conjugated antibodies were used: PE Mouse anti‐SNAI2/Slug (BD Biosciences, 564615), Alexa Fluor 488 Mouse anti‐human Sox17 (BD Biosciences, 562205), PerCPCy5.5 Mouse anti‐human PAX6 (BD Biosciences, 562388), Alexa Fluor 488 mouse anti‐MAP2B (BD Biosciences, 560399), PE Mouse anti‐human Sox1 (BD Biosciences, 561592), Alexa Fluor 647 mouse anti‐Oct3/4 (BD Biosciences, 560253), V450 mouse anti‐Sox2 (BD Biosciences, 561610), PE‐Cy7 Mouse anti‐human CD34 (BD Biosciences, 560710), Alexa Fluor 488 mouse anti‐SOX17 (BD Biosciences 562205), PE mouse anti‐FOXA2 (BD Biosciences, 561589) Alexa Flour 488 mouse anti‐Oct3/4 (BD Biosciences, 560253), Alexa Fluor 647 anti‐Nanog (BD Biosciences, 561300), PerCP‐Cy5.5 mouse anti‐Tra‐1‐81 (BD Biosciences, 561575).

### 4.11 Genetic stability

The copy number of 8 chromosomes commonly gained or lost in pluripotent cells was quantified using the hPSC Genetic Analysis kit (StemCell Technologies Cat.#07550) in accordance with the manufacturer's instructions. qPCR was run on LightCycler480 (Roche). Copy number was calculated relative to chromosome 4 of NDF cells using the formula 2*2^(‐2ΔΔCT)^.

### 4.12 Sendai virus expression analysis

The presence of sendai virus was assessed using primer sequences from the CytoTune 2.0 kit. Total RNA was extracted from passage 7 cell pellets using the RNeasy Kit (Quiagen Cat. #74104) and quantified using Nanodrop. 750 ng RNA was used for cDNA synthesis using the iScript RT Master mix (Cat). cDNA was diluted 1:5 and 3 μL of each diluted sample was used for each PCR reaction. SeV was amplified using FWD primer GGATCACTAGGTGATATCGAGC and REV primer ACCAGACAAGAGTTTAAGAGATATGTATC, KOS was amplified using FWD primer ATGCACCGCTACGACGTGAGCGC and REV primer ACCTTGACAATCCTGATGTGG. PCR reactions contained 0.4 μM of each primer, 0.2 mM dNTPs 1X PCR buffer and 1.25 U of HotStar Taq (Quiagen Cat.# 203205). Cycling conditions were 95C × 15 min, 35 cycles of 95C × 0.5 min, 55C × 0.5 min, 72C × 0.5 min and a final extension at 72C for 10 min for SeV and 95C × 15 min, 35 cycles of 95C × 0.5 min, 60C × 0.5 min, 72C × 0.5 min and a final extension at 72C for 10 min for KOS.

### 4.13 Directed differentiation

Directed differentiation was performed using the STEMdiff Trilineage Differentiation Kit (StemCell Technologies Cat.# 05230) according to the manufacturer's instructions. Briefly, iPSCs were harvested and plated as single cells in mTeSR Plus containing 10 μM Y‐27632 at a density of 8 × 105 cells per well in a 12 well plate for ectoderm and endoderm differentiation, and at 2 × 105 cells per well in a 12 well plate for mesoderm differentiation. To account for the decreased growth rate of Δ4977 mtDNA mutant iPSCs, cells were plated at 1 × 106 cells per well for ectoderm and endoderm differentiation, and at 3 × 105 cells per well for mesoderm differentiation. One day following plating, the media was changed to lineage specific media and changed daily thereafter. Mesoderm and endoderm differentiation wells were harvested on the fifth day following addition of lineage specific media, and ectoderm differentiation wells were harvested on the seventh day following media addition.

### 4.14 Immunocytochemistry of iPSC‐derived cardiomyocytes

For imaging, iPSC‐derived cardiomyocytes were harvested using TrypLE Select 10 (Gibco, Cat# A1217701), grown for 3 days, fixed in 4% formaldehyde for 10 min and then stained with anti‐Cardiac Troponin T (Abcam ab45932) and anti‐alpha‐actinin (Sigma Aldrich A7811) at 1:800 in accordance with previously described methods (Martirosian et al., 2021). Samples were imaged on a Leica SP8 Confocal Microscope using the LAS X software. Negative control samples were imaged first to adjust microscope settings and account for possible background staining. Z‐stack images were taken at 63× using system‐optimized intervals. Line and Frame image information was averaged eight times. Acquired images were further processed using the Lightning algorithm and normalized using Photon Count.

### 4.15 Autocorrelation quantification of Cardiomyocyte beating

Video images were collected on a Canon EOS Rebel T3 Digital SLR camera (DS126291, Canon) at 30 Hz. Videos were decomposed into individual frames using custom MATLAB (MathWorks) scripts and ran through an 2D autocorrelation function. Resultant autocorrelation output was further processed using custom MATLAB (MathWorks) scripts and MATLAB polyfit function (MathWorks) to extract peaks per minute as our measure of cardiomyocyte beating.

### 4.16 Quantitative phase imaging

Quantitative phase imaging (QPI) of iPSC and MPC was performed on an Axio Observer.A1 inverted microscope (Zeiss) in a temperature and CO_2_ regulated stage‐top cell incubation chamber at 20× magnification and 0.40 numerical aperture objective. Interferogram images were collected every 10 min for up to 48 h data using a quadriwave lateral shearing interferometry (QWLSI) (Bon et al., 2009) camera (SID4BIO, Phasics) with illumination provided by a 660 nm center wavelength collimated LED (Thorlabs). Image processing, segmentation, total mass per cell, and mass accumulation rate measurements were done through custom MATLAB (MathWorks) scripts as described previously (Nguyen et al., 2020).

### 4.17 RNA sequencing

iPSCs were grown to 80% confluency and RNA was extracted using the RNeasy Mini Kit (Quiagen) and RNase‐free DNase (Quiagen) following the manufacturer's protocol. Quality control was performed using TapeStation. Sequencing libraries were prepared by the UCLA Technology Center for Genomics & Bioinformatics using the TruSeq stranded mRNA protocol. Paired‐end sequencing was performed at 2 × 150 bp on a NovaSeq XP. Reads were quality filtered and converted into fastq files, then quasi‐mapped and quantified to the Gencode Homo Sapiens GRCh38 all cDNA reference transcriptome using Salmon (v1.9.0) (Patro et al., 2017) in python 3.9.15. Differential gene expression analysis and gene set enrichment analysis were conducted in the R environment (R v 4.2). Normalized transcript counts were extracted and differential gene expression analysis was performed using DESeq2 (v1.38.1) (Love et al., 2014). Significance testing was performed using Wald test, resulting P‐values were adjusted for multiple testing using package default. Gene set enrichment analysis was performed using the fgsea package (v1.24.0) and reactome pathways from reactome.db. Pathways with NES magnitude greater than 1.5 and at least 10 included transcripts were considered.

### 4.18 Metabolomics

Extraction for cellular metabolites and ultra‐high performance liquid chromatography and mass spectrometry (UHPLC ‐MS) sample analysis was performed as previously described (Patananan et al., 2020). Briefly, cells were rinsed with ice‐cold 150 mM NH4AcO, pH 7.3 followed by quenching with ice‐cold 80% methanol with 1 nmol norvaline internal standard added for 1 h at −80°C. Cells were scraped and contents were transferred to a 1.5 mL Eppendorf tube then vortexed for 10 s and centrifuged at 16,000*g*× 15 min at 4°C. The supernatant was transferred into a glass vial, dried down without heat in a Genevac EZ‐2 Elite evaporator, and resuspended in 50% acetonitrile: water and one‐tenth of the resuspension was injected for UHPLC–MS processing. Metabolites were separated by a Luna NH2 (150 × 2 mm) column by Phenomenex using 5 mM NH4AcO, pH 9.9 as solvent A and acetonitrile as solvent B. Gradient performance was as follows: 15% solvent A to 95% solvent A over 18 min, with 9 min isocratic flow and re‐equilibration to 15% solvent A. Metabolites were analyzed on a Vanquish Flex UPLC (Thermo Scientific) coupled to a Q Exactive mass spectrometer (Thermo Scientific) operating on polarity switching (+3.50 kV/−3.50 kV) in full scan mode with a 70–975 m/z range. Data was processed using Maven (Open Source; v8.1.27.11). Intensities were normalized by protein pellet amount quantified by BCA protein assay (ThermoFisher, Cat. # 23225).

### 4.19 Metabolomics analysis

Metabolite abundance was normalized to presence of the standard metabolite, norvaline. Samples were processed in two batches, each with even numbers of wildtype and mutant sample. To normalize between runs, data from each run was auto‐scaled by centering each metabolite on the metabolite mean and dividing by the metabolite standard deviation before combining run data for further analysis. Principle component and differential metabolite presence analysis was using the statistical language R4.4.2 and Bioconductor v3.15 packages. For pathway‐level enrichment analysis, the mean of each metabolite for each sample was used to control for difference in number of technical replicates per sample. Enrichment analysis was performed using MetaboAnalyst5.0 web server (Pang et al., 2021) using the smpdb pathway database with the standard full metabolite set as background.

### 4.20 Lipidomics

Shotgun lipidomics were performed through the UCLA Lipidomics Core via direct infusion‐tandem mass spectrometry on the CIEX 5500 tripl‐quadrupole (QQQ) with a Shimadzu auto‐sampler configured for direct infusion experiments, SelexION ion mobility device, and Shimadzu LC. Data is analyzed using a previously described in‐house workflow and a combination of 80 lipid standards sourced from Sciex and Avanti Polar lipids (Su et al., 2021).

### 4.21 Epigenetic age measurement

DNA was extracted from cell pellets using Quiagen DNeasy according to manufacturer's protocol and concentrated using AMPure beads. To control for potential passage impact, all iPSC clones were analyzed at passage 9 following establishment of pluripotency. DNA was bisulfite converted and methylation was measured using the Infinium Epic V2 array in accordance with manufacturer's specifications. Analysis was conducted in the R environment (R v 4.2)t IDAT files were processed using *Minfi* (v1.44.0) (Fortin et al., 2017), normalization was done using *preprocessNoob*. Epigenetic age was calculated using dnaMethyAge v0.2.0 (Wang et al., 2023) to calculate the “HorvathS2013” age.

## AUTHOR CONTRIBUTIONS

AV and MAT planned the study and wrote the manuscript. AV, AS, AT performed cell culture, reprogramming and differentiation. AV and JG performed DNA extraction, genotyping. TN performed LCI and LCI analysis, MD performed metabolite harvest. AV performed metabolomic and RNA sequencing data analysis. VM and AS performed ICC. AH and JW performed nanopore sequencing.

## FUNDING INFORMATION

ARV was supported by NIH 1K08AG086582‐01, the Dermatology Foundation and the Melanoma Research Alliance. MTD is supported by the UCLA Graduate Division Dr. Ursula Mandel Scholarship and the California Institute for Regenerative Medicine UCLA Eli and Edythe Broad Center of Regenerative Medicine and Stem Cell Research Training Program. MAT supported by NIH award P30CA016042 to the UCLA Jonsson Comprehensive Cancer Center. TLN is supported by the iCMB NIH T32 Post‐doctoral Training Program. AS is supported by EDUC2‐08411CSUN CIRM Bridges 3.0 Stem Cell Research & Therapy Training Program. A.T. acknowledges the support of the UCLA Eli and Edythe Broad Center of Regenerative Medicine and Stem Cell Research Rose Hills Foundation Graduate Scholarship Training Program. V.M. acknowledges the support of the UCLA Eli and Edythe Broad Center of Regenerative Medicine and California Institute of Regenerative Medicine.

## CONFLICT OF INTEREST STATEMENT

The authors have nothing to disclose.

## Supporting information


Figure S1.

Table S1.

Table S2.

Table S3.

## Data Availability

RNA sequencing data is available through SRA Bioproject PRJNA1019386. Raw metabolomic and lipidomic data are available from the PI by request.
