# Supplementary material for: Increased mitochondrial mutation heteroplasmy induces aging phenotypes in pluripotent stem cells and their differentiated progeny
Source: Aging Cell. 2024 Dec 16;24(3):e14402. doi: 10.1111/acel.14402 (PMC11896400; doi:10.1111/acel.14402)
Supplement: Supplementary file 1 — Figure S1. [file ACEL-24-e14402-s001.docx]

**Supplemental Figures**


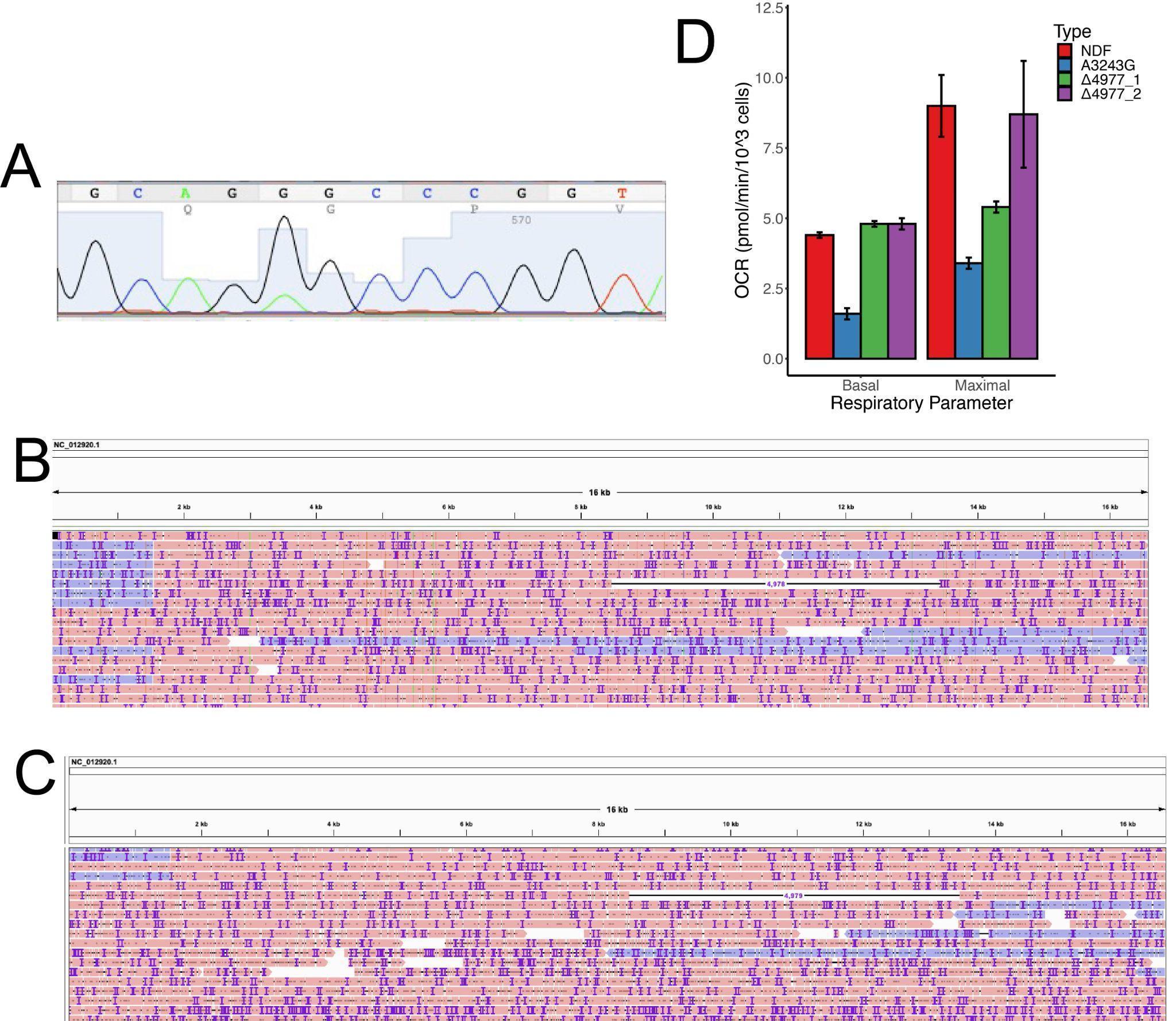


**Supplemental Figure 1: Characterization of fibroblasts used for reprogramming.** A) Sanger sequencing of A3243G mutant fibroblasts. Shown are chromosome M, bases 3239-325. B-C) Nanopore sequencing of chromosome M from Δ4977 fibroblasts. Shown are IGV alignment maps of full length chromosome M reads from Δ4977 fibroblasts line 1 (B) and line 2 (C).D) Basal and maximal oxygen consumption rate over time per 10^3 cells as measured by Seahorse extracellular flux assay.


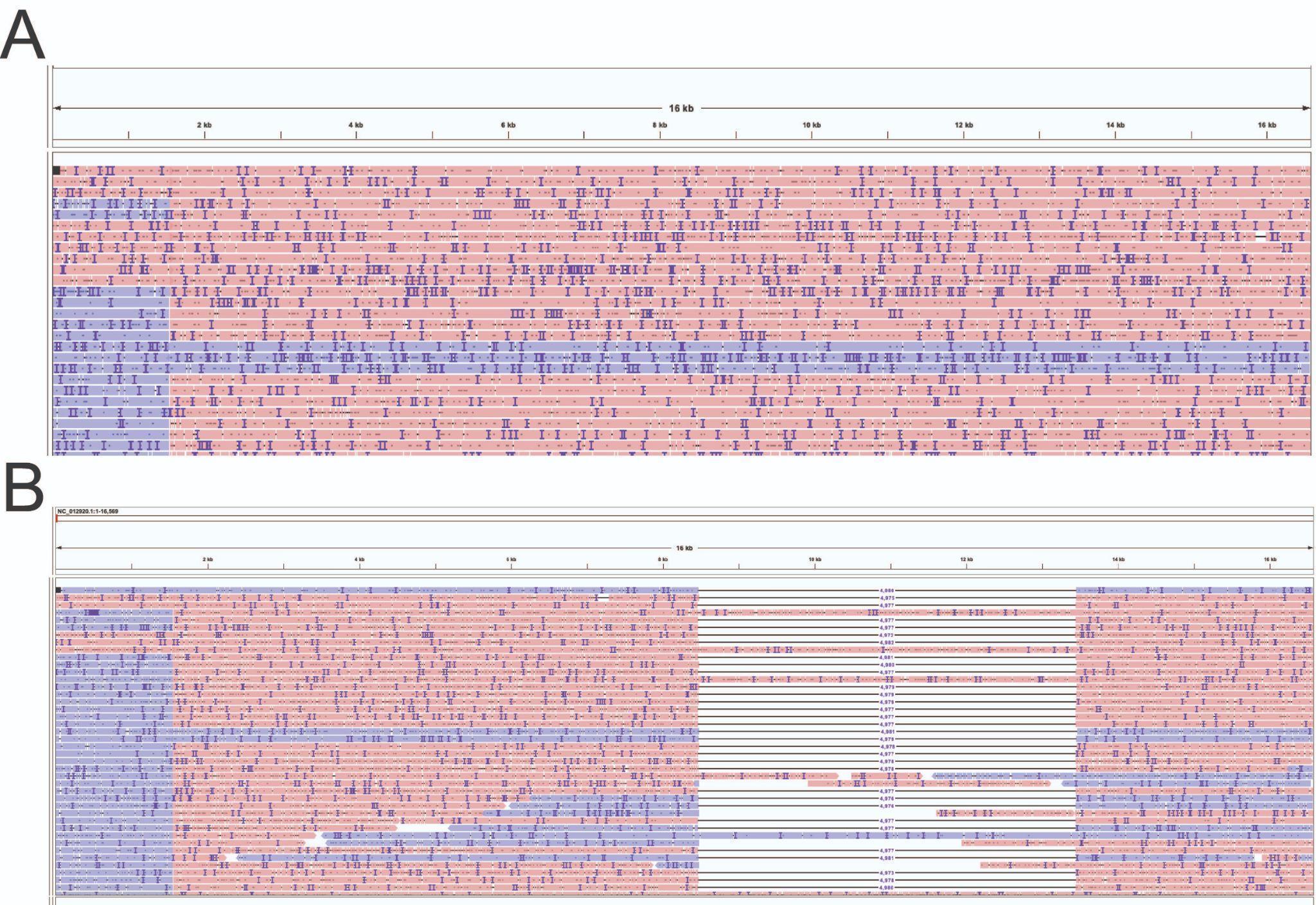


**Supplemental Figure 2: Validation of genotype after reprogramming.** Shown are Integrated Genome Viewer (IGV) alignment maps of full length chromosome M reads iPSC with no d4977 mutation on ddPCR (A) and iPSC with high d4977 heteroplasmy on ddPCR (B).


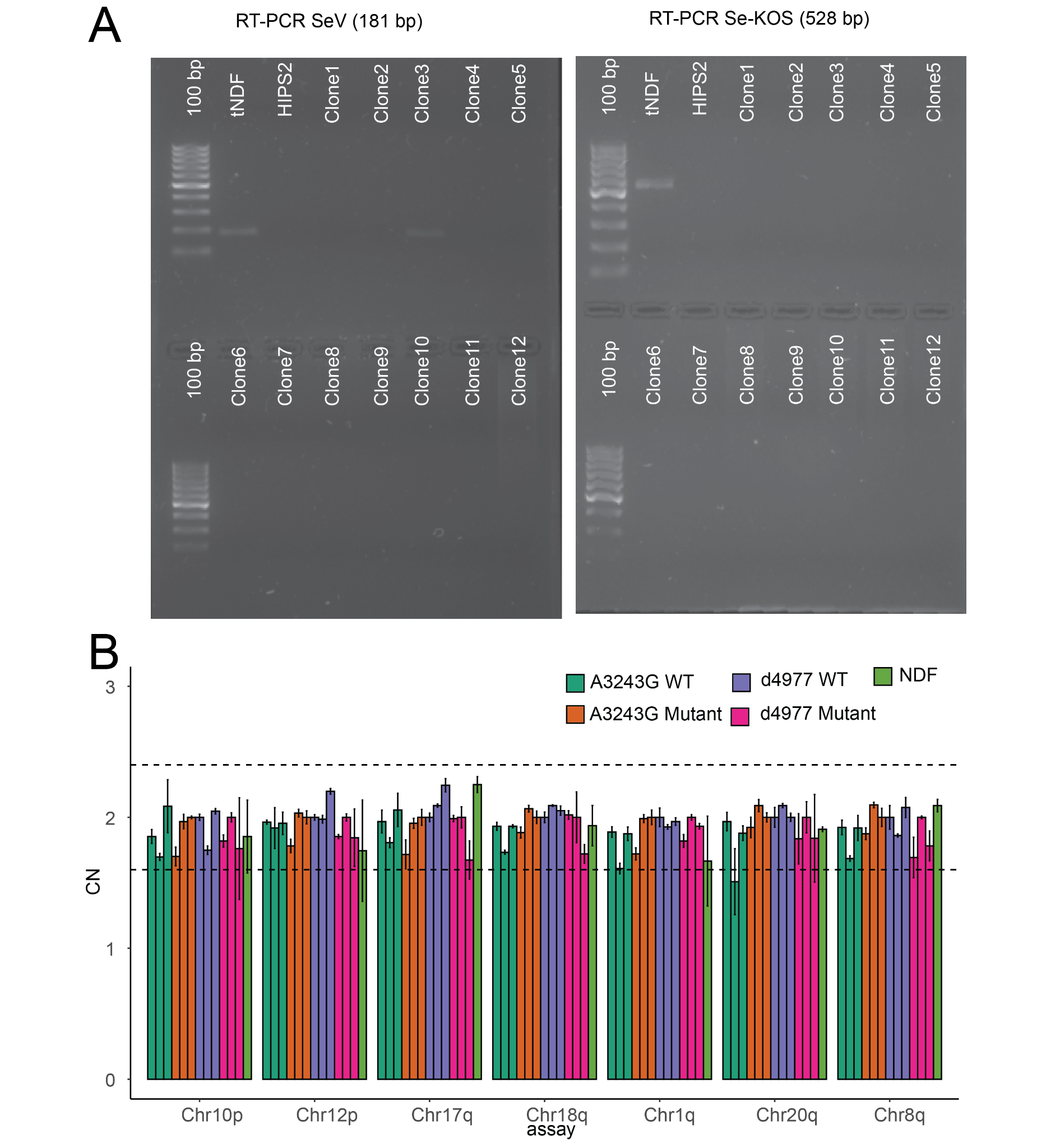


**Supplemental Figure 3: Characterization of iPSC clones.** A) PCR amplification of viral vector RNA from recently transfected fibroblasts (tNDF), control iPSC line (HIPS2) and examined iPSC clones. B) qPCR aneuploidy screening of iPSC lines using Stem Diff HPSC Genetic Analysis kit to examine copy number of 7 chromosomes in iPSC clones and NDF fibroblast line.


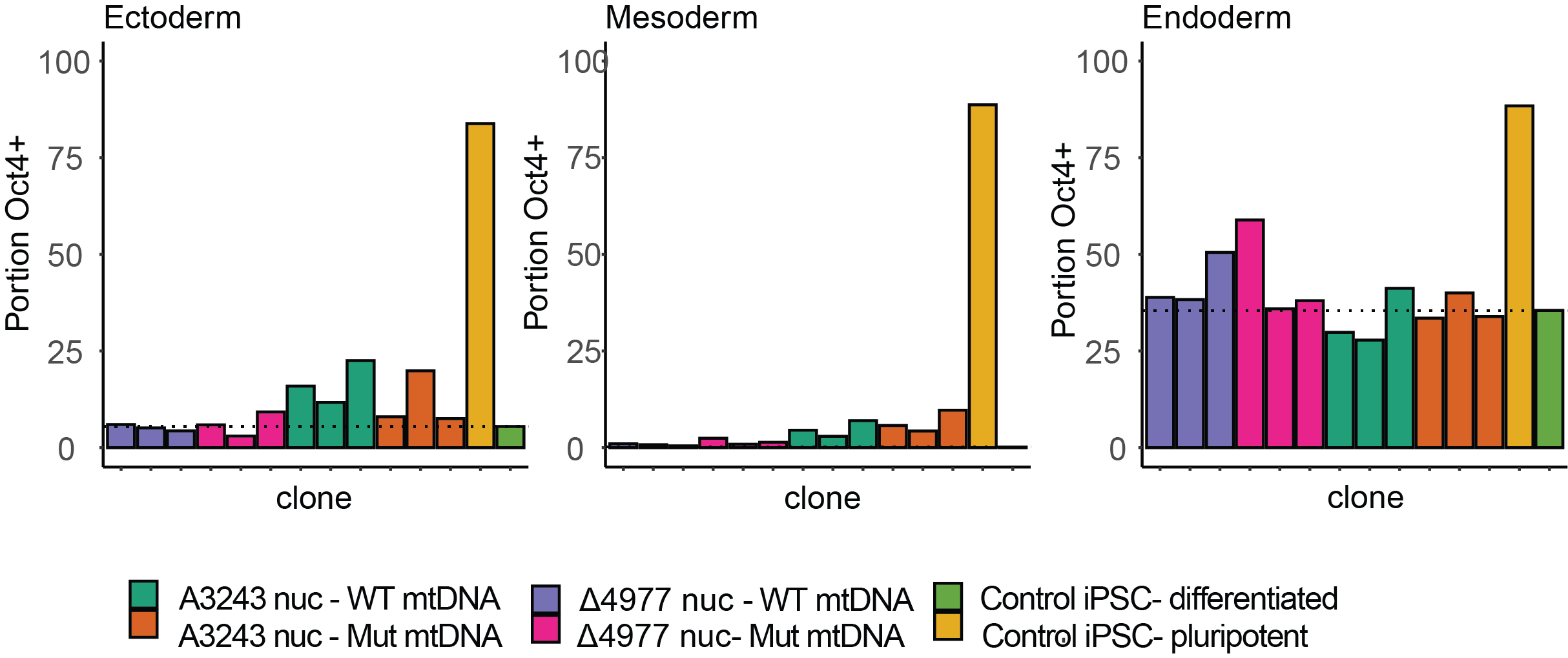


**Supplementary Figure 4: Loss of pluripotency with trilineage differentiation.** Percentage of putative lineage progenitor cells derived from each iPSC line expressing Oct4+ measured by flow cytometry.


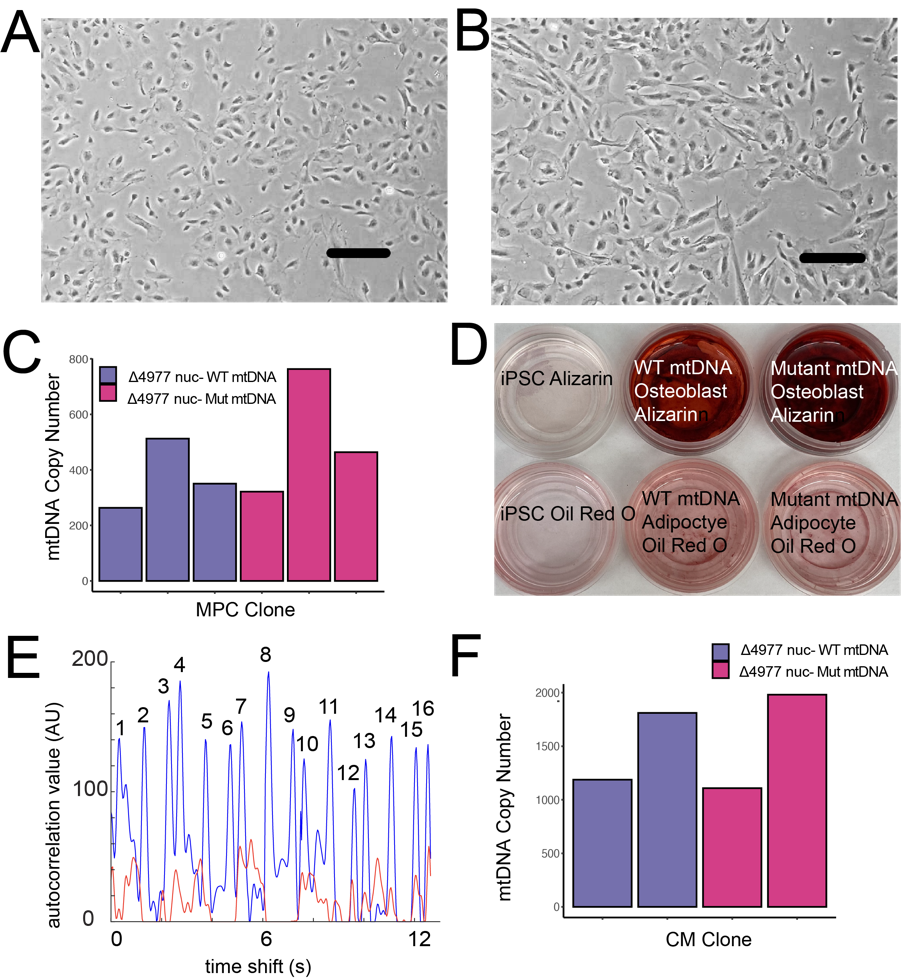


**Supplemental Figure 5: Terminal Differentiation of mtDNA mutant iPSC.** **A)** Morphology of WT mtDNA iPSC derived mesenchymal progenitor cells. Scale bar= 100 uM. **B)** Morphology of high d4977 mtDNA iPSC derived mesenchymal progenitor cells. Scale bar= 100 uM. **C)** DdPCR quantification of mtDNA copy number in MPC lines derived from WT mtDNA and d4997 mtDNA iPSC clones. **D)** Osteogenesis and adipogenesis of iPSC derived MPC. Top row: Alizarin red staining of calcium crystals from iPSC (first column) and WT mtDNA osteoblasts (second column) and d4997 mtDNA osteoblasts (third column). Bottom row: Oil red O staining of lipid droplets from iPSC (first column) and WT mtDNA adipocytes (second column) and d4997 mtDNA adipocytes (third column). **E)** Quantification of contraction of iPSC-derived cardiomyocytes, those derived from mtDNA WT iPSC shown in blue, those derived from high d4997 mtDNA iPSC shown in red. **F)** DdPCR quantification of mtDNA copy number in cardiomyoctes derived from WT mtDNA and d4997 mtDNA iPSC clones.


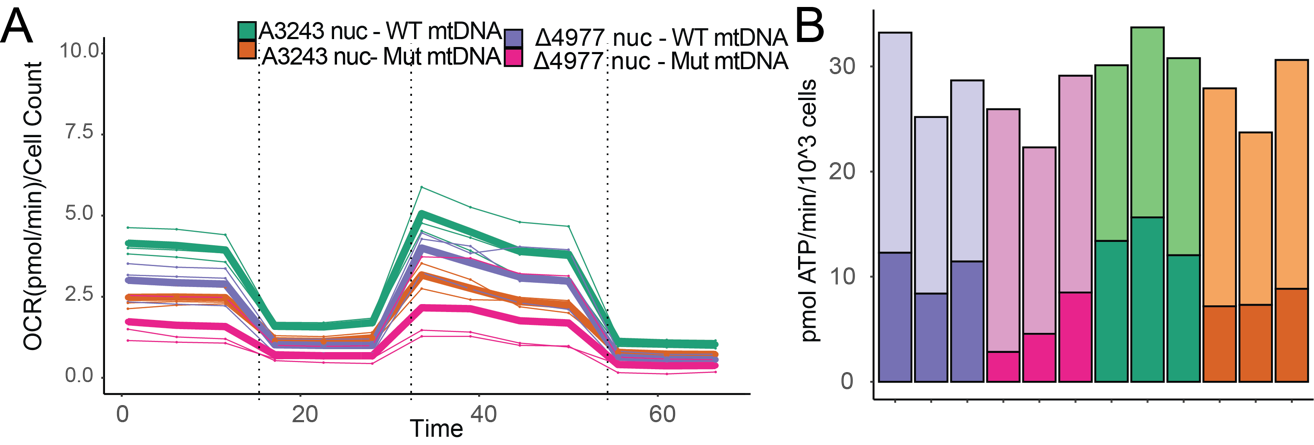


**Supplemental Figure 6: Reduced oxidative metabolism in mtDNA mutant iPSCs.** A) Oxygen consumption rate over time per 10^3 cells as measured by Seahorse extracellular flux assay. Each thin line shows the mean tracing for five technical replicates, and each thick line shows the mean of three biological replicates for each group. B) Estimated ATP generated by oxidative phosphorylation (dark lower bars) and glycolysis (lighter bars) for each set of iPSC clones as measured by Seahorse extracellular flux assay. Shown are means for five technical replicates per clone.


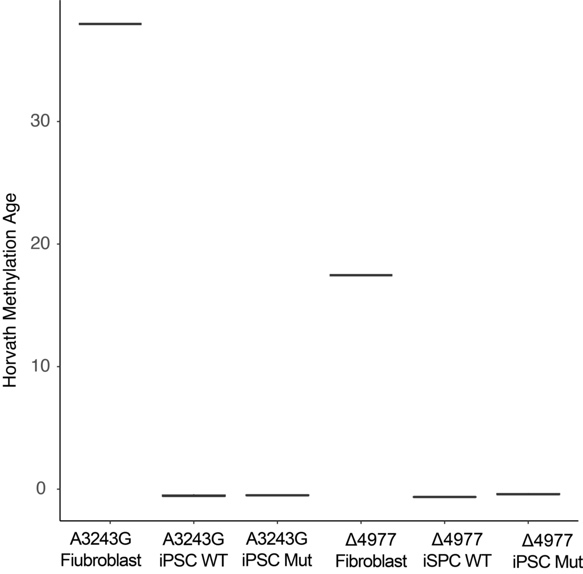


**Supplemental Figure 7:** **DNA methylation age is variably reverted with reprogramming to pluripotency.** Shown is the DNA methylation age calculated using the Horvath 2013 formula for parental fibroblasts and iPSC clones containing either absent or high mtDNA mutation heteroplasmy.
